# Isolation and Identification of Plant Growth Promoting Rhizobacteria from Cucumber Rhizosphere and Their Effect on Plant Growth Promotion and Disease Suppression

**DOI:** 10.3389/fmicb.2015.01360

**Published:** 2016-02-02

**Authors:** Shaikhul Islam, Abdul M. Akanda, Ananya Prova, Md. T. Islam, Md. M. Hossain

**Affiliations:** ^1^Department of Plant Pathology, EXIM Bank Agricultural UniversityChapainawabganj, Bangladesh; ^2^Department of Biotechnology, Bangabandhu Sheikh Mujibur Rahman Agricultural UniversityGazipur, Bangladesh; ^3^Department of Plant Pathology, Bangabandhu Sheikh Mujibur Rahman Agricultural UniversityGazipur, Bangladesh

**Keywords:** PGPR, plant growth promotion, IAA production, biological nitrogen fixation, antagonism, *Phytophthora capsici*, disease suppression

## Abstract

Plant growth promoting rhizobacteria (PGPR) are the rhizosphere bacteria that may be utilized to augment plant growth and suppress plant diseases. The objectives of this study were to identify and characterize PGPR indigenous to cucumber rhizosphere in Bangladesh, and to evaluate their ability to suppress Phytophthora crown rot in cucumber. A total of 66 isolates were isolated, out of which 10 (PPB1, PPB2, PPB3, PPB4, PPB5, PPB8, PPB9, PPB10, PPB11, and PPB12) were selected based on their *in vitro* plant growth promoting attributes and antagonism of phytopathogens. Phylogenetic analysis of 16S rRNA sequences identified these isolates as new strains of *Pseudomonas stutzeri*, *Bacillus subtilis*, *Stenotrophomonas maltophilia*, and *Bacillus amyloliquefaciens*. The selected isolates produced high levels (26.78–51.28 μg mL^-1^) of indole-3-acetic acid, while significant acetylene reduction activities (1.79–4.9 μmole C_2_H_4_ mg^-1^ protein h^-1^) were observed in eight isolates. Cucumber plants grown from seeds that were treated with these PGPR strains displayed significantly higher levels of germination, seedling vigour, growth, and N content in root and shoot tissue compared to non-treated control plants. All selected isolates were able to successfully colonize the cucumber roots. Moreover, treating cucumber seeds with these isolates significantly suppressed Phytophthora crown rot caused by *Phytophthora capsici*, and characteristic morphological alterations in *P. capsici* hyphae that grew toward PGPR colonies were observed. Since these PGPR inoculants exhibited multiple traits beneficial to the host plants, they may be applied in the development of new, safe, and effective seed treatments as an alternative to chemical fungicides.

## Introduction

The cucumber (*Cucumis sativus*) is one of the most widely grown vegetable crops in the world, and is particularly prevalent on the Indian sub-continent. This crop is prone to massive attacks by *Phytophthora capsici* that causes crown rot and blight (Kim et al., 2008; Maleki et al., 2011). *P. capsici* infects susceptible hosts throughout the growing season at any growth stage, and causes yield losses as high as 100% (Lee et al., 2001). This pathogen has a wide host range with more than 50 plant species including Cucurbitaceae, Leguminosae, and Solanaceae (Hausbeck and Lamour, 2004). Although fungicides can control the disease, their use is detrimental to the surrounding environment and to the viability and survival of beneficial rhizosphere microbes (Carson et al., 1962; Hussain et al., 2009; Heckel, 2012). Furthermore, the growing cost of pesticides and the consumer demand for pesticide-free food have led to a search for substitutes for these products. Thus, there has been a need to find effective alternatives to costly and environmentally degrading synthetic pesticides.

Rhizobacteria that benefit plants by stimulating growth and suppressing disease are referred to as plant growth promoting rhizobacteria (PGPR; Kloepper et al., 1980). PGPR have been tested as biocontrol agents for suppression of plant diseases (Gerhardson, 2002), and also as inducers of disease resistance in plants (Cattelan et al., 1999; Bargabus et al., 2002; Bais et al., 2004). In particular, strains of *Pseudomonas*, *Stenotrophomonas*, and *Bacillus* have been successfully used in attempts to control plant pathogens and increase plant growth (Bais et al., 2004; Idris et al., 2007; Liu et al., 2007; Messiha et al., 2007; Chen et al., 2009; El-Sayed et al., 2014). The widely recognized mechanisms of plant growth promotion by PGPR are production of phytohormones, diazotrophic fixation of nitrogen, and solubilization of phosphate. Mechanisms of biocontrol action include competition with phytopathogens for an ecological niche or substrate, as well as production of inhibitory compounds and hydrolytic enzymes that are often active against a broad spectrum of phytopathogens (Zhang and Yuen, 2000; Manjula et al., 2004; Haas and Défago, 2005; Stein, 2005; Detry et al., 2006; Konsoula and Liakopoulou-Kyriakides, 2006; Cazorla et al., 2007).

Many PGPR have been shown to reduce Phytophthora crown rot occurrence on various plants. Ahmed et al. (2003) demonstrated *in vitro* suppression of *P. capsici* by bacterial isolates from the aerial part and rhizosphere of sweet pepper. An endophytic bacterium isolated from black pepper stem and roots, *B. megaterium* IISRBP17 suppressed *P. capsici* on black pepper in greenhouse assays (Aravind et al., 2009). Zhang et al. (2010) demonstrated that PGPR strains used separately or in combinations had the potential to suppress Phytophthora blight on squash in the greenhouse. Shirzad et al. (2012) reported that some fluorescent pseudomonads isolated from different fields of East and West Azarbaijan and Ardebil provinces of Iran exhibited strong antifungal activity against *P. drechsleri* and controlled crown and root rot of cucumber caused by the pathogen. However, little is known about PGPRs with the potential to suppress crown rot caused by *P. capsici* on cucumber. Furthermore, the plant-growth-promoting and biocontrol efficacy of PGPR often depend upon the rhizosphere competence of the microbial inoculants (Lugtenberg and Kamilova, 2009). Rhizosphere competence refers to the survival and colonization potential of PGPR (Bulgarelli et al., 2013), and is thought to be highest for each PGPR strain when associated with its preferred host plant. This to some extent explains why some PGPR strains exhibiting promise as biocontrol agents *in vitro* have variable biocontrol efficacy in the rhizosphere of a given crop under a given set of conditions. The identification and characterization of PGPR populations indigenous to cucumber rhizospheres is therefore critical to discovery of strains that can be utilized to improve growth and Phytophthora crown rot suppression in cucumber. The objectives of the present study were to isolate bacterial strains from the cucumber rhizosphere, to characterize these isolates on the basis of morphological and physiological attributes as well as by 16S rRNA sequence analysis, and to assess the plant growth promoting effects of these isolates *in vivo* and their ability to suppress Phytophthora crown rot in cucumber plants. To our knowledge, this is the first report of PGPR reducing *P. capsici* infection on cucumber.

## Materials and Methods

### The Study Site

The experimental site was located at the Field Laboratory of the Department of Plant Pathology, Bangabandhu Sheikh Mujibur Rahman Agricultural University (BSMRAU), Gazipur, Bangladesh. The location of the site is at 24.09° N latitude and 90.25° E longitude with an elevation of 2–8 m. The study area is within the Madhupur Tract agro-ecological zone (AEZ 28). The soil used for pot experiments belongs to the Salna series and has been classified as “swallow red brown terrace soil” in the Bangladesh soil classification system, which falls under the order Inceptisol (Brammer, 1978). This soil is characterized by clay within 50 cm of the surface and is slightly acidic in nature. The pH value, cation exchange capacity (CEC) and electrical conductivity (EC) of bulk soil samples collected from the study site were 6.38, 6.78 meq 100 g^-1^ soil and 0.6 dS m^-1^, respectively. This soil contained 1.08% organic carbon (OC), 1.87% organic matter (OM), 0.10% nitrogen (N), 9 ppm phosphorus (P) and 0.20 meq 100 g^-1^ soil exchangeable potassium (K).

### Plant Material, Bacterial Isolation, and Pathogenic Organism

Cucumber (*Cucumis sativus* L.) variety Baromashi (Lal Teer Seed Company, Dhaka, Bangladesh) root samples were collected from the study site along with rhizosphere soil. For isolation of bacteria, 2–5 *g* of fresh roots were washed under running tap water and surface sterilized in 5% NaOCl for 1 min. After washing three times with sterilized distilled water (SDW), the root samples were ground with a sterilized mortar and pestle. Serial dilutions were prepared from the ground roots, and 100 μl aliquots from each dilution of 1 × 10^-6^, 1 × 10^-7^, and 1 × 10^-8^ CFU mL^-1^ were spread on potato dextrose agar (PDA) plates and incubated for 2 days at 28 ± 2°C. Morphologically distinct bacterial colonies were selected for further purifications. The purified isolates were preserved temporarily in 20% glycerol solution at -20°C. The pathogen *P. capsici* was provided by Prof. W. Yuanchao, Nanjing Agricultural University, China.

### Morphological and Biochemical Characterization of Bacterial Isolates

Colony morphology, size, shape, color, and growth pattern were recorded after 24 h of growth on PDA plates at 28 ± 2°C as described by Somasegaran and Hoben (1994). Cell size was observed by light microscopy. The Gram reaction was performed as described by Vincent and Humphrey (1970). A series of biochemical tests were conducted to characterize the isolated bacteria using the criteria of Bergey’s Manual of Systematic Bacteriology (Bergey et al., 1994). For the KOH solubility test, bacteria were aseptically removed from Petri plates with an inoculating wire loop, mixed with 3% KOH solution on a clean slide for 1 min and observed for formation of a thread-like mass. The motility of each isolate was tested in sulfide indole motility (SIM) medium. Using a needle, strains were introduced into test tubes containing SIM, and were incubated at room temperature until the growth was evident (Kirsop and Doyle, 1991). Turbidity away from the line of inoculation was a positive indicator of motility. Catalase and oxidase tests were performed as described in Hayward (1960) and Rajat et al. (2012), respectively. To determine whether the rhizobacterial isolates are better suited to aerobic or anaerobic environments, the citrate test was conducted according to Simmons (1926) using Simmons citrate agar medium. All experiments were done following complete randomized design (CRD) with three replications for each isolate and repeated once.

### Molecular Characterization of Bacterial Isolates

Culture DNA was obtained using the lysozyme-SDS-phenol/chloroform method (Maniatis et al., 1982). DNA was extracted with phenol-chloroform-isoamyl alcohol (25:24:1) and precipitated with isopropanol. The extracted DNA was treated with DNase-free RNase (Sigma Chemical Co., St. Louis, MO, USA) at a final concentration of 0.2 mg/ml at 37°C for 15 min, followed by a second phenol-chloroform-isoamyl alcohol extraction and isopropanol precipitation. Finally, the DNA pellet was re-suspended in TE buffer (10 mM Tris-HCl, 1 mM EDTA, pH 8.0), stored at -20°C, and used as template DNA in PCR to amplify the 16S rRNA for phylogenetic analysis.

16S rRNA gene amplification was performed by using the bacterial-specific primers, 27F (5^′^-AGAGTTTGATCCTGGCTCAG-3^′^) and 1492R (5^′^-GGTTACCTTGTTACGACTT-3^′^) (Reysenbach et al., 1992). PCR amplifications were performed with 1 × Ex Taq buffer (Takara Bio Inc, Japan), 0.8 mM dNTP, 0.02 units μl^-1^ Ex Taq polymerase, 0.4 mg ml^-1^ BSA, and 1.0 μM of each primer. Three independent PCR amplifications were performed at an annealing temperature of 55°C (40 s), an initial denaturation temperature of 94°C (5 min), 30 amplification cycles with denaturation at 94°C (60 s), annealing (30 s), and extension at 72°C (60 s), followed by a final extension at 72°C (10 min). The PCR product was purified using Wizard^^®^^ PCR Preps DNA Purification System (Promega, Madison, WI, USA). Purified double-stranded PCR fragments were directly sequenced with Big Dye Terminator Cycle sequencing kits (Applied Biosystems, Forster City, CA, USA) using the manufacturer’s instructions. Sequences for each region were edited using Chromas Lite 2.0^1^. The 16S rRNA sequence of the isolate has been deposited in the GenBank database. The BLAST search program^2^ was used to search for nucleotide sequence homology for the 16S region for bacteria. Highly homologous sequences were aligned, and neighbor-joining trees were generated using ClustalX version 2.0.11 and MEGA version 6.06. Bootstrap replication (1000 replications) was used as a statistical support for the nodes in the phylogenetic trees.

### Bioassays for Plant Growth Promoting Traits

#### Biological Nitrogen Fixation

Nitrogenase activity of isolates was determined via the acetylene reduction assay/ethylene production assay as described in Hardy et al. (1968). Pure bacterial colonies were inoculated to an airtight 30 ml vial containing 10 ml nitrogen-free basal semi-solid medium, and were grown for 48 h at 28 ± 2°C. Following pellicle formation, the bottles were injected with 10% (v/v) acetylene gas and incubated at 28 ± 2°C for 24 h. Ethylene production was measured using a G-300 Gas Chromatograph (Model HP 6890, USA) fitted with a Flame Ionization Detector and a Porapak-N column. Hydrogen and oxygen were used as a carrier gas, with a flow rate of 4 kg/cm^2^, and the column temperature was maintained at 165°C. The ethylene concentration calibration curve was plotted for each trial, and the viable cell numbers (cfu) of the isolate were determined. The rate of N_2_ fixation was expressed as the quantity of ethylene accumulated (μmol C_2_H_4_ mg^-1^ protein h^-1^) based on the standard curve and peak-area percentage.

#### Indole-3-Acetic Acid Production

For detection and quantification of indole-3-acetic acid (IAA) production by bacterial isolates, isolated colonies were inoculated into Jensen’s broth (Sucrose 20 g, K_2_HPO_4_ 1 g L^-1^, MgSO_4_ 7H_2_O 0.5 g L^-1^, NaCl 0.5 g L^-1^, FeSO_4_ 0.1 g L^-1^, NaMoO_4_ 0.005 g L^-1^, CaCO_3_ 2 g L^-1^) (Bric et al., 1991) containing 2 mg mL^-1^ L-tryptophan. The culture was incubated at 28 ± 2°C with continuous shaking at 125 rpm for 48 h (Rahman et al., 2010). Approximately 2 mL of culture solution was centrifuged at 15000 rpm for 1 min, and a 1 mL aliquot of the supernatant was mixed with 2 mL of Salkowski’s reagent and incubated 20 min in darkness at room temperature (150 ml concentrated H_2_SO_4_, 250 ml distilled water, 7.5 ml 0.5 M FeCl_3_.6H_2_O) as described by Gordon and Weber (1951). IAA production was observed as the development of a pink-red color, and the absorbance was measured at 530 nm using a spectrophotometer. The concentration of IAA was determined using a standard curve prepared from pure IAA solutions (0, 5, 10, 15, 20, 25, 30, 35, 40, 45, 50, 55, 60, and 65 μg ml^-1^).

### Preparation of Bacterial Inocula for Cucumber Seed Treatment

Bacterial strains were cultured in 250 ml conical flasks containing 200 ml yeast peptone broth on an orbital shaker at 120 rpm for 72 h at 28 ± 2°C. Bacterial cells were collected via centrifugation at 15000 rpm for 1 min at 4°C, and each pellet was washed twice with SDW. The bacterial pellets were suspended in 0.6 ml SDW, vortexed and used for seed treatment. Approximately 30–31 cucumber seeds were surface sterilized in 5% NaOCl for 1 min and washed three times in SDW. Dry seeds were immersed in each bacterial suspension, and the preparation was stirred frequently for 5 min. The treated seeds were spread on a petri dish and air dried overnight at room temperature. The number of bacterial cells per seed, determined via serial dilutions, was approximately 10^8^ CFU/seed.

### Effect of Bacterial Seed Treatment on Germination and Vigour Index in Cucumber

In order to determine the effect of the isolates on germination and seedling vigour, 100 seeds inoculated with each isolate were incubated in ten 9-cm petri dishes on two layers of moistened filter paper. As a control treatment, seeds treated with water instead of bacterial suspensions were also established. In order to maintain sufficient moisture for germination, 5 ml distilled water was added to the petri dishes every other day, and seeds were incubated at 28 ± 2°C in a light incubator. Germination was considered to have occurred when the radicles were half of the seed length. The germination percentage was recorded every 24 h for 7 days. Root and shoot length were measured after the seventh day. The experiment was planned as a completely randomized design with 10 replications for each isolate.

$$Ger\min ation\,\;rate\,\;\;\;(\%)=(\frac{number\,\;\;of\,\;\;seeds\,\;\;ger\,\min ated}{total\,\;\;number\,\;\;of\,\;\;seeds})\times 100$$

         Vigour⁢  index=%   germination×total⁢  plant⁢  length

### Effect of Bacterial Seed Treatment on Growth and Nitrogen Content in Cucumber Plants

In order to test the ability of isolates to promote growth in cucumber plants, surface-sterilized cucumber seeds were inoculated with each isolate as described above. The soil from the study site described above was used as potting medium. After autoclaving twice at 24 h intervals at 121°C and 15 psi for 20 min, 180 g of the sterilized soil was placed in each sterilized plastic pot (9.5 cm × 7.0 cm size). One pre-germinated cucumber seed was sown in each pot, and plants were grown 3 weeks in a net house with watering on alternate days. After harvest, the fresh weight, dry weight, and root and shoot lengths of the plants were measured. The shoots and roots were separated and dried in an oven at 68 ± 2°C for 48 h, then ground for determination of tissue-N concentrations (Kjeldahl, 1883).

### Root Colonization

Root colonization by bacterial isolates was determined according to the protocol of Hossain et al. (2008). Roots were harvested from plants at 7, 14, and 21 days of growth. Root systems were thoroughly washed with running tap water to remove adhering soil particles, then were rinsed three times with SDW and blotted to dryness. Roots were divided into top, middle, and bottom regions, and were weighed and homogenized in SDW. Serial dilutions were prepared on PDA plates, and the number of colony-forming units (cfu) per gram root was determined after 24 h of incubation at 28 ± 2°C.

### Pathogenicity of *P. capsici* in Cucumber

For preparation of zoospore inoculum, *P. capsici* was cultured on PDA plates at 18 ± 2°C for 7 days. Five-mm blocks were then cut from the culture plates and placed in petri dishes containing SDW. The petridishes were incubated in darkness at room temperature for 72 h, followed by a 1-h cold treatment at 4°C. Zoospore production was confirmed via light microscopy. In order to test the pathogenicity of *P. capsici* zoospores, cucumber seedlings were planted in pots containing 0, 500, 1000, or 1500 μl zoospores/pot. As 100% mortality was found in case 1000 μl zoospores/pot, two concentrations (500 and 1000 μl) of zoospores suspension per pot were fixed.

### *In Vitro* Screening for Antagonism

To test antagonism of *P. capsici* by each isolate, the pathogen and bacteria were inoculated 3 cm apart on the same agar plate. Fungal growth on each plate was observed, and the zone of inhibition, if present, was determined as described in Riungu et al. (2008):

$$\%Inhibition\,\;\;\;of\,\;\;my\,\;\;mycelial\,\;\;growth=\frac{X-Y}{X}\times 100$$

Where,

X = Mycelial growth of pathogen in absence of antagonists

Y = Mycelial growth of pathogen in presence of antagonists

Morphologies of hyphae in the vicinity of bacterial colonies were observed under a light microscope (Meiji Techno: ML2600), and images were recorded with a digital camera (Model: Canon Digital IXUS 900 Ti). Each experiment was carried out following CRD with three replications for each isolate and repeated once.

### Testing the Effect of Rhizobacterial Seed Treatment on Phytophthora Crown Rot of Cucumber

Cucumber seeds inoculated with each isolate were sown and grown for 7 days in sterilized plastic pots as described above. Seven-day-old seedlings were inoculated with 500 or 1000 μl zoospore suspension/pot as described in Deora et al. (2005). Inoculated plants were kept inside humid chambers for 48 h. Each experiment included 12 plants per treatment with three replications. Surviving plants were counted 7 days after inoculation. Percent disease incidences (PDI) were calculated using the following formula:

$$P\,D\,I=\frac{N\,u\,m\,b\,e\,r\,\;\;o\,f\,\;\;\inf e\,c\,t\,e\,d\,\;\;p\,l\,a\,n\,t\,s}{T\,o\,t\,a\,l\,\;\;\;n\,u\,m\,b\,e\,r\,\;\;o\,f\,\;\;i\,n\,o\,c\,u\,l\,a\,t\,e\,d\,\;\;p\,l\,a\,n\,t\,s}\times 100\%$$

Percent protection by PGPR was calculated using following formula:

$$\%\;protection=\left[\frac{A-B}{A}\right]\times 100\%$$

Where,

A = PDI in non-inoculated control plants

B = PDI in PGPR-treated plants.

### Statistical Analysis

Statistical analyses were performed using SPSS (Version 17) and Microsoft Office Excel 2007. A completely randomized design was used for all experiments, with 3–12 replications for each treatment. The data presented are from representative experiments that were repeated at least twice with similar results. Treatments were compared via ANOVA using the least significant differences test (LSD) at 5% (*P ≤* 0.05) probability level.

## Results

### Strain Isolation and Biochemical and Molecular Characterization

We obtained a total of 66 rhizobacterial strains from the interior of cucumber roots. Ten isolates – PPB1, PPB2, PPB3, PPB4, PPB5, PPB8, PPB9, PPB10, PPB11, and PPB12 – were selected based on their ability to produce IAA, fix N_2_, and show *in vitro* antagonism against various pathogens in a preliminary screening. All isolates were rods producing fast-growing, round to irregular colonies with raised elevations and smooth surfaces. Reddish pigmentation was produced by PPB5, while no pigmentation was produced by other isolates (Supplementary Table S1). All 10 isolates were motile and reacted positively to the Gram staining, citrate, catalase and oxidase tests, but reacted negatively to the KOH solubility test (**Table 1**).

**Table 1:** Biochemical and molecular analysis of the endophytic bacterial isolates.

Strains	Biochemical analysis							Molecular analysis
	KOH Test	Gram reaction	Citrate Test	Catalase Test	Oxidase Test	IAA (μg/ml)	ARA (μmole C_2_H_4_ mg protein/h)	Identification based on 16S rRNA gene sequencing
PPB1	**-**	**+**ve	**+**	**+**	**+**	39.67 ± 0.12^∗^	0.00 ± 0.00^∗^	*Pseudomonas stutzeri*
PPB2	**-**	**+**ve	**+**	**+**	**+**	41.43 ± 0.71	4.90 ± 0.23	*Bacillus subtilis*
PPB3	**-**	**+**ve	**+**	**+**	**+**	26.78 ± 0.69	4.55 ± 0.38	*Stenotrophomonas maltophilia*
PPB4	**-**	**+**ve	**+**	**+**	**+**	50.18 ± 0.23	2.41 ± 0.12	*B. amyloliquefaciens*
PPB5	**-**	**+**ve	**+**	**+**	**+**	51.28 ± 0.41	2.90 ± 0.17	*B. subtilis* subsp. *subtilis*
PPB8	**-**	**+**ve	**+**	**+**	**+**	44.41 ± 0.22	4.79 ± 0.01	*B. subtilis*
PPB9	**-**	**+**ve	**+**	**+**	**+**	41.75 ± 0.93	3.95 ± 0.02	*B. subtilis* subsp. *spizizenii*
PPB10	**-**	**+**ve	**+**	**+**	**+**	38.43 ± 0.82	1.83 ± 0.01	*B. amyloliquefaciens*
PPB11	**-**	**+**ve	**+**	**+**	**+**	40.30 ± 0.23	1.79 ± 0.01	*B. subtilis* subsp. *subtilis*
PPB12	**-**	**+**ve	**+**	**+**	**+**	29.25 ± 0.97	0.00 ± 0.00	*B. amyloliquefaciens*

*‘-’ corresponds to negative response; ‘+ve’ and ‘+’ correspond to positive responses. ^∗^Values are the Mean ± SE. The experiment was repeated twice with three replicates for each isolate*.

Phylogenetic trees constructed from 16S rRNA sequences showed that the selected isolates were mainly members of genus *Bacillus, Pseudomonas*, and *Stenotrophomonas* (Supplementary Figure S1). The sequences of the isolates PPB2, PPB5, PPB8, PPB9, and PPB11 showed 99% similarity with *Bacillus subtilis* and were submitted to GenBank under accession numbers KJ690255, KM008605, KM008606, KM092525 and KM092527, respectively (**Table 1**). Isolate PPB1 had 99% homology with *Pseudomonas stutzeri* and was submitted to GenBank under accession number KJ959616. Isolate PPB3 was identified as *Stenotrophomonas maltophilia* with GenBank accession number KJ959617. Isolates PPB4, PPB10, and PPB12 showed 99% sequence homology with *B. amyloliquefaciens* and were submitted to GenBank under accession number KM008604, KM092526 and KM092528, respectively (**Table 1**).

### Characterization for Plant Growth Promoting Traits

The plant growth promoting characteristics viz., IAA production and ARA were examined with the ten selected PGPR isolates. The results of the assays are presented in **Table 2**. In the presence of tryptophan, the isolated bacteria produced IAA in concentrations between 26.78 μg mL^-1^ and 51.28 μg mL^-1^. The highest and lowest amounts of IAA were produced by strain PPB5 and PPB3, respectively (**Table 2**). Nitrogenese activity, as determined by ARA, was not detected in PPB1 and PPB12 under the conditions tested. However, the ARA values ranged from 1.79 to 4.9 μmole C_2_H_4_/mg protein/h for remaining isolates. PPB2 showed the highest activity, while the lowest was recorded for PPB11 (**Table 1**). The other isolates also reduced acetylene in significant amounts. Collectively, these results suggest that the isolates possess a number of traits associated with plant growth promotion.

**Table 2:** Comparative performance of PGPR in mycelia growth inhibition of *P. capsici* and Phytophthora crown rot disease suppression in cucumber plants.

Treatments	Pathogen suppression^a^ (% *P. capsici* mycelial growth inhibition)	Disease suppression^b^ (% Protection^c^)	


		500 μl Zoospores/pot^d^	1000 μl Zoospores/pot
PPB1	67.16 ± 0.68e*	58.33 ± 0.66c	33.33 ± 2.18b
PPB2	70.01 ± 0.85g	69.45 ± 0.80e	38.84 ± 1.70c
PPB3	69.08 ± 0.91f	50.00 ± 1.69b	50.00 ± 1.54e
PPB4	62.07 ± 0.11c	70.33 ± 2.37f	45.68 ± 2.37d
PPB5	65.94 ± 0.53d	50.00 ± 0.57b	33.33 ± 1.69b
PPB8	82.05 ± 0.55j	83.33 ± 0.56h	77.78 ± 2.25g
PPB 9	90.08 ± 0.46k	66.67 ± 1.87d	66.67 ± 2.93f
PPB10	73.08 ± 0.83h	73.67 ± 1.53g	66.67 ± 0.52f
PPB11	58.32 ± 0.12b	88.83 ± 1.67i	86.08 ± 2.23h
PPB12	80.53 ± 0.69i	50.00 ± 1.15b	33.33 ± 0.43b
Control	0.00 ± 0.00a	0.00 ± 0.00a	0.00 ± 0.00a

*^∗^Values are the means ± SE (*n = 12*). Data within the same column followed by different letters are significantly different*.

*^a^Pathogen suppression was measured as percent inhibition of radial growth of *P. capsici* by antagonistic PGPR in dual plate assay*.

*^b^Disease suppression was expressed as percent protection due to treatment with PGPR relative to control (non-inoculated)*.

*^c^Protection (%) = [(A - B)/A] × 100 in which, A = PDI in non-inoculated control plants and B = PDI in PGPR-treated plants*.

*^d^Seven-day-old seedlings were inoculated with 500 or 1000 μl zoospore suspension/pot*.

*The data presented are from representative experiments that were repeated twice with similar results*.

### Germination and Vigour Index Improvement in Cucumber

**FIGURE 1:**
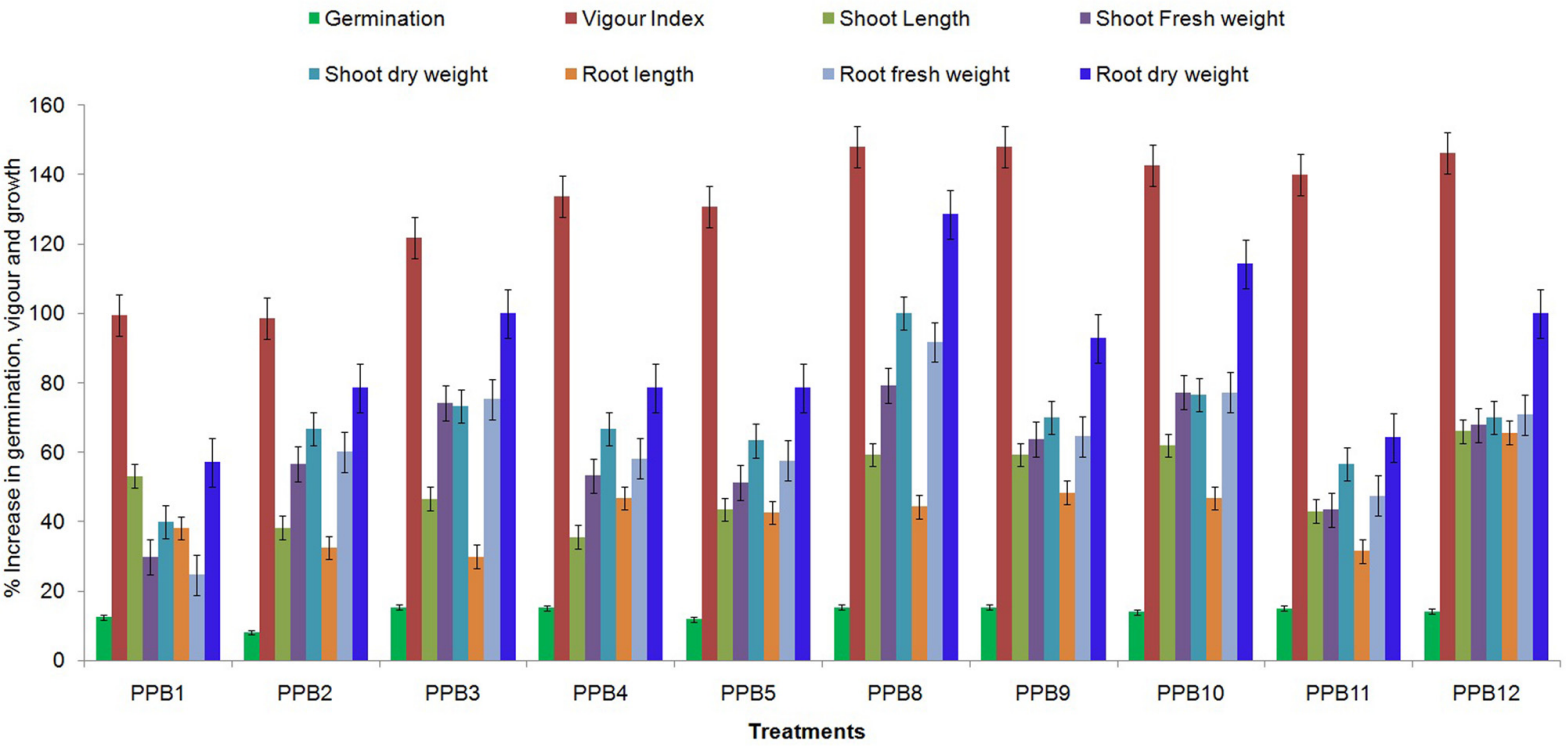
**Effect of plant growth promoting rhizobacteria (PGPR) treatments on seed germination, vigour and growth characteristics of cucumber seedlings grown in pots under axenic conditions**. Error bars are SE from three replicates per same treatment. Data are presented as % increase in germination, vigour index, shoot length, root length, shoot fresh weight, root fresh weight, shoot dry weight, and root dry weight of PGPR-treated cucumber seedlings relative to non-treated control seedlings. The experiment was repeated twice.

The effect of rhizobacterial treatment upon seed germination and vigour index of cucumber varied with different isolates. All treatments had a significant effect on the germination rate and vigour index compared to the control. The PGPR treatments increased the germination rate of cucumber seeds by 8.07–15.32% compared with the control, while the vigour index was increased by 98.62–148.05% (**Figure 1**). In both germination rate and vigour index, the maximum increase was obtained with the PPB9 treatment. These results suggest that rhizobacterial treatment could improve the germination and vigour of cucumber seeds.

### Plant Growth Promotion in Cucumber

All isolates significantly increased the growth of cucumber compared to non-inoculated controls. Treatment with isolate PPB12 produced the maximum shoot and root lengths of 18.23 and 20.47 cm, corresponding to increases of 66.02 and 65.63% above control treatments (**Figure 1**). However, the maximum shoot and root weight enhancement was observed in PPB8-treated plants. Treatment with isolate PPB8 produced shoot fresh and dry weights of 5.29 and 0.60 g plant^-1^, which were 79.32 and 100.00% higher than those of control plants. Similarly, treatment with isolate PPB8 produced root fresh and dry weights of 3.03 and 0.32 g plant^-1^, corresponding to increase of 91.77 and 128.57% above control treatments.

### N Concentration in Cucumber Plants

**FIGURE 2:**
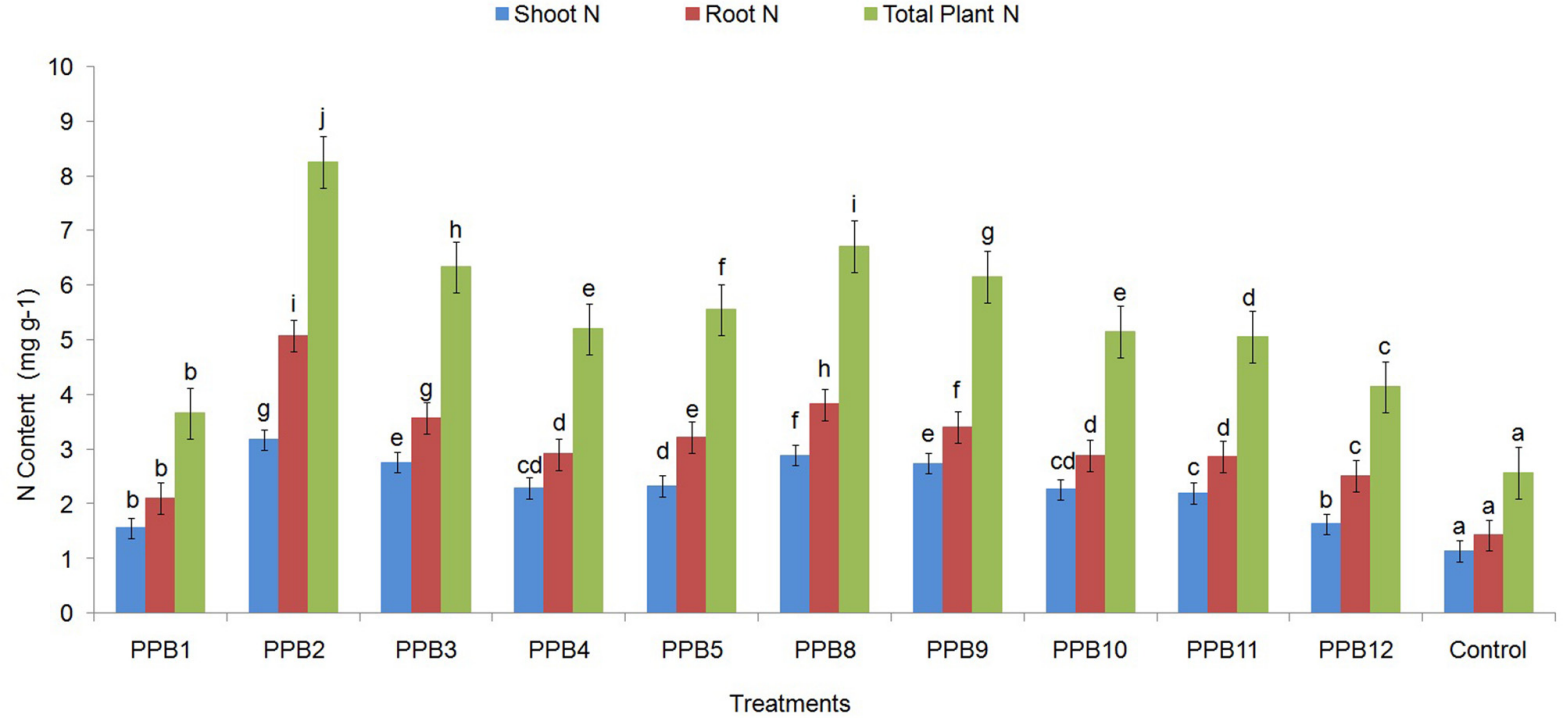
**Effect of inoculation with PGPR strains on shoot and root N contents of cucumber plants**. Error bars are SE from three replicates that received the same treatment. Data represents total shoot and root N concentration (mg g^-1^), each from 3 sets of 8–10 shoots and roots sampled following harvesting the cucumber plants. Within each frame different letters indicate statistically significant difference between treatments (LSD test, *P ≤ 0.05*). The experiment was repeated twice.

The N content in plant roots and shoots significantly increased due to inoculation treatments with rhizobacterial isolates (**Figure 2**). The shoot and root N content showed similar trends in response to different treatments; hence, the N content is reported as the total combined shoot and root N. The total N content in PGPR-treated plants ranged from 3.66 mg g^-1^ to 8.25 mg g^-1^ N compared with 2.57 mg g^-1^ N for non-inoculated control plants, a 42–221% increase in PGPR-treated plants over control plants (**Figure 2**). The highest N content was recorded in plants grown under PPB2 followed by PPB8, PPB3, PPB9 and other treatments.

### Root Colonization

**FIGURE 3:**
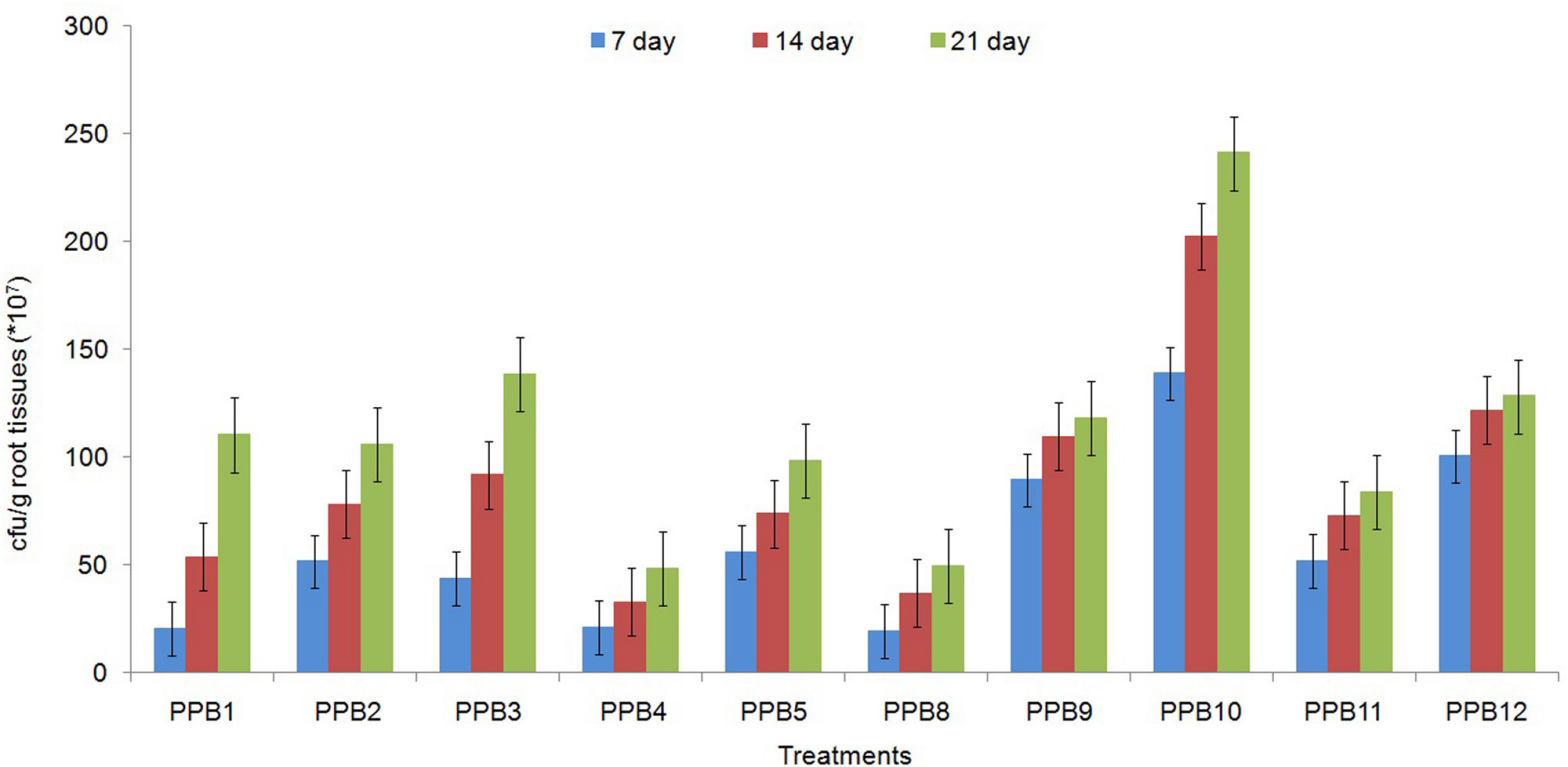
**Population density (cfu) of different PGPR strains from roots of 7-, 14-, and 21-day-old cucumber seedlings**. Error bars are SE from three replicates per treatment. Data are presented as numbers of c.f.u. g^-1^ fresh weight, each from three sets of 5–8 whole roots. The data presented are from representative experiments that were repeated twice with similar results.

The ability to colonize the root system is essential for rhizobacteria to be effective plant growth promoters. The root colonization assays showed that all the tested isolates successfully colonized the roots of cucumber plants as tested after only 7 days of seedling growth. The inoculated populations were even higher on 21-day-old roots. Nevertheless, the root population densities varied widely among the isolates (**Figure 3**). The largest root populations were observed for strain PPB2, followed by PPB5 and PPB9 (**Figure 3**). These results demonstrate specific interactions between cucumber plants and the rhizobacterial isolates.

### *In vitro* Antagonism of *Phytophthora capsici*

**FIGURE 4:**
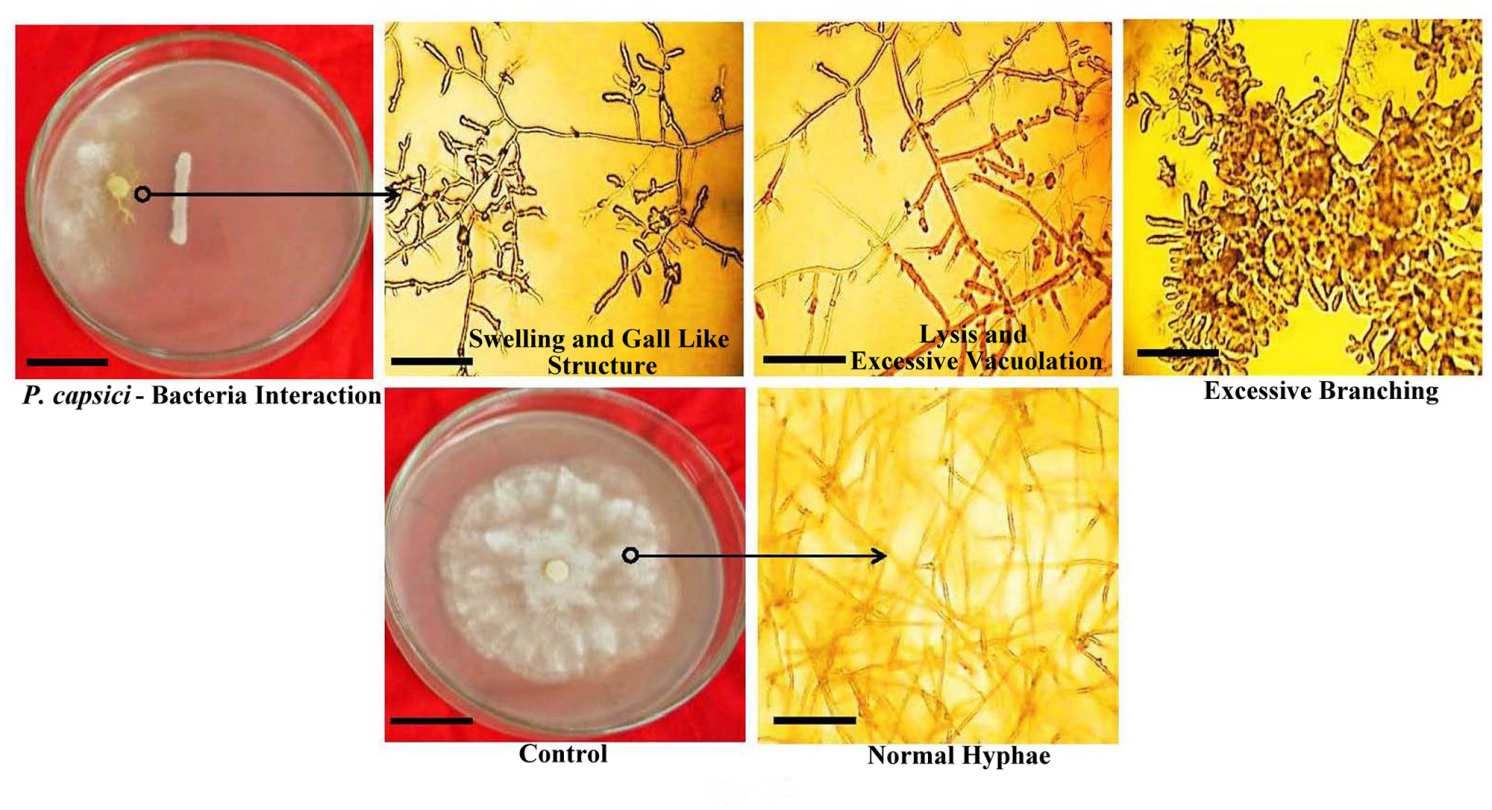
***In vitro* interactions of PGPR strains with *P. capsici* on PDA plates, including morphological alterations in *P. capsici* hyphae**.

All rhizobacterial isolates exhibited significant antagonistic activity against *P. capcisi* on PDA. The largest inhibition zone was observed with PPB9 (90.08%) followed by PPB8 (82.05%) (**Table 2**). Distinct morphological alterations in *P. capcisi* hyphae were also detected during dual cultures with the rhizobacterial isolates. Hyphal features observed in the vicinity of bacterial colonies included irregular and excessive branching, abnormal swelling of hyphal diameters, unusually long and pointed hyphal tips, and vacuolization leading to hyphal lysis (**Figure 4**).

### Suppression of Phytophthora Crown Rot in Cucumber

All the selected PGPR strains showed consistent suppression of Phytophthora crown rot in the greenhouse experiments. Compared with the control, the average disease protection at 500 μl zoospore suspension ranged from 50 to 88.83% after treatment with rhizobacterial isolates, while protection at 1000 μl zoospore suspension ranged from 33.33 to 86.08% (**Table 2**). At both inoculum rates, isolate PPB11 showed the highest disease reduction, and the lowest disease reduction was obtained with PPB5.

## Discussions

PGPR colonizing the surface or inner part of roots play important beneficial roles that directly or indirectly influence plant growth and development (Glick et al., 1999; Gerhardt et al., 2009). In this study, 10 PGPR classified as *Pseudomonas stutzeri* (PPB1), *B. subtilis* (PPB2, 5, 8, 9, and 11), *S. maltophilia* (PPB3), and *B. amyloliquefaciens* (PPB4, 10, and 12) were isolated from the rhizosphere of cucumber plants. All the isolated PGPR were gram positive and motile rods, and tested positive for the ability to utilize citrate as a carbon source. Flagellar motility and citrate utilization are both thought to play a significant role in competitive root colonization and maintenance of bacteria in roots (Turnbull et al., 2001; Weisskopf et al., 2011). These strains also tested positive for oxidase and catalase activity. Standard microbiology references suggest that *S. maltophilia* is an oxidase-negative bacterium (Ryan et al., 2009). Recent data, however, suggest that some *S. maltophilia* are oxidase-positive (Carmody et al., 2011), and this was also the case for isolate PPB3 in this study. Our catalase test results corroborate prior studies showing that *B. subtilis*, *Pseudomonas* stutzeri, and *B. amyloliquefaciens* are catalase-positive (Merchant and Packer, 1999; Kamboh et al., 2009). *Bacillus* and *Pseudomonas* are the most frequently reported genera of PGPR (Laguerre et al., 1994; Hallmann and Berg, 2006; Zahid et al., 2015). Similarly, most isolates in this study belong to genus *Bacillus*.

Treatment of cucumber seeds with the selected isolates significantly improved seedling emergence and growth. Several different mechanisms have been suggested for similar observations using other PGPR strains: PGPR might indirectly enhance seed germination and vigour index by reducing the incidence of seed mycoflora, which can be detrimental to plant growth (Begum et al., 2003). Duarah et al. (2011) found that amylase activity during germination was increased in rice and legume after inoculation with PGPR. The amylase hydrolyzes the starch into metabolizable sugars, which provide the energy for growth of roots and shoots in germinating seedlings (Beck and Ziegler, 1989; Akazawa and Nishimura, 2011). One of the most commonly reported mechanisms is the production of phytohormones such as IAA (Patten and Glick, 2002). All the selected isolates in this study produced IAA. Similar studies have shown that IAA production is very common among PGPR (Yasmin et al., 2004; Ng et al., 2012; Zahid et al., 2015). In fact, many isolates in this study produced higher IAA than previously reported strains (Yasmin et al., 2004; Banerjee et al., 2010; Ng et al., 2012). This is an important mechanism of plant growth promotion because IAA promotes root development and uptake of nutrients (Carrillo et al., 2002). It has long been proposed that phytohormones act to coordinate demand and acquisition of nitrogen (Kiba et al., 2011). Therefore, enhanced N-content found in inoculated plants could be due to increased N-uptake by the roots caused by hormonal effects on root morphology and activity. Nitrogen fixation may also play a role in plant growth promotion. All the selected isolates in this study except PPB1 and PPB12 showed acetylene reduction activity, which is a widely accepted surrogate for nitrogenase activity and N_2_-fixing potential (Andrade et al., 1997). However, defensible proof of N_2_-fixation needs the application of ^15^N as tracer of soil N or as ^15^N_2_-gas and the demonstration of significantly changed N-isotope-labeling in the plant biomass. Transfer of N between diazotrophic N-fixing rhizobacteria and the roots of several crops has been demonstrated (Islam et al., 2009; Abbasi et al., 2011; Tajini et al., 2012; Verma et al., 2013). It is interesting to note that in this study all isolates, including the two that demonstrated no acetylene reduction activity, enhanced the N content of cucumber. This suggests that while N_2_ fixation may be an important mechanism of plant growth promotion, there may also be alternate mechanisms, like hormonal interactions and nutrient uptake or pathogen suppression, which might be more pronounced than the contribution of nitrogen fixation.

Results from our study indicate that PGPR strains applied as a seed treatment significantly reduced disease severity of Phytophthora crown rot on cucumber plants. The fungal antagonists *Pseudomonas stutzeri*, *B. subtilis*, *B. amyloliquifaciens*, and *S. maltophilia* were have been shown to be effective biocontrol agents in prior studies (Dunne et al., 2000; Zhang and Yuen, 2000; Dal Bello et al., 2002; Berg et al., 2005; Islam and Hossain, 2013; Erlacher et al., 2014). Competitive root tip colonization by PGPR strains might play an important role in the efficient control of soil-borne diseases. The crucial colonization level that must be reached has been estimated at 10^5^–10^6^ cfu g^-1^ of root in the case of *Pseudomonas* sp., which protect plants from *Gaeumannomyces tritici* or *Pythium* sp. (Haas and Défago, 2005). Most of our selected strains were efficient colonizers of roots, since CFU counts for tested strains were more than 10^7^ cfu g^-1^ root. However, the former study examined the root colonization by introduced bacteria under natural field soil conditions, while our study did under axenic conditions. In view of that, comparison between root colonization data obtained under these two conditions may not be accurate. Biological control of *P. capsici* can also result from antibiosis by the bacteria (Nakayama et al., 1999; Kawulka et al., 2004; Chung et al., 2008; Lim and Kim, 2010; Mousivand et al., 2012; Islam and Hossain, 2013). All the selected isolates exhibited moderate to high antagonistic activity against *P. capsici in vitro*, and caused clear morphological distortions such as abnormal branching, curling, swelling and lysis of the hyphae at the interaction zone in dual cultures. Excessive branching and curling accompanied by marked ultrastructural alterations including invagination of the hyphal membrane, disintegration or necrosis of hyphal cell walls, accumulation of excessive lipid bodies, enlarged and electron-dense vacuoles, and degradation of cytoplasm were potentially due to bacterial production of antibiotics and lytic enzymes (Deora et al., 2005; Islam and von Tiedemann, 2011). These antibiotics and lytic enzymes cause membrane damage and are particularly inhibitory to zoospores of Oomycete (de Souza et al., 2003; Beneduzi et al., 2012). Induced systemic resistance is most likely another mechanism by which bacteria suppress *P. capsici* (Zhang et al., 2010).

In the present study, we have isolated 10 new strains of PGPR from indigenous cucumber plants. These strains possessed several plant growth promoting traits as well as antifungal activity, and were found to be efficient in controlling Phytophthora crown rot of cucumber seedlings. *In vitro* and *in vivo* evidence suggest that the selected isolates benefit cucumber plants via multiple modes of action including antibiosis against phytopathogens, competitive colonization, and plant growth promotion. This reveals the potential of these strains for biofertilizer applications and commercial use as biocontrol agents in the field. However, from the estimation of a PGPR-potential to a biofertilizer application, it requires a long way of greenhouse experiments with pot filled with different type of soils and finally, field experiments to find out the optimum formulations for the inoculums. Thus, the inoculants can perform close to its optimum potential. Future studies concerning commercialization and field applications of integrated stable bio-formulations as effective biocontrol strategies are in progress.

## Author Contribution

SI was involved in the planning and execution of the research work; collection, analysis and interpretation of the data; manuscript writing etc. following the suggestions and directions of the Major Professor. AMA served as the Member of the Dissertation Committee of SI and was involved in the planning of the work and editing of the manuscript. AP was actively involved in the original research work, data collection, analysis as well as manuscript preparation. TI supplied the *Phytophthora capsici* inocula and oversaw the sequence work of the bacterial isolates and related description in the manuscript. MMH served as the Major Professor of SI and was involved in the research design and planning; analysis and interpretation of data; drafting as well as critical revision of the work for intellectual content.

All authors approve the final version of the manuscript for publication and agrees to be accountable for all aspects of the work in ensuring that questions related to the accuracy or integrity of any part of the work are appropriately investigated and resolved.

## Conflict of Interest Statement

The authors declare that the research was conducted in the absence of any commercial or financial relationships that could be construed as a potential conflict of interest.

## Footnotes

1 http://www.technelysium.com.au/chromas.html.

2 http://blast.ncbi.nlm.nih.gov/Blast.cgi

## Supplementary Material

FIGURE S1 **| Phylogenetic tree of 16S rRNA gene sequences showing the relationships among the isolates isolated from cucumber rhizosphere**. The data of type strains of related species were from GenBank database.
