# Supplementary figures and images for: Isolation and Identification of Plant Growth Promoting Rhizobacteria from Cucumber Rhizosphere and Their Effect on Plant Growth Promotion and Disease Suppression
Source: Front Microbiol. 2016 Feb 2;6:1360. doi: 10.3389/fmicb.2015.01360 (PMC4735380; doi:10.3389/fmicb.2015.01360)

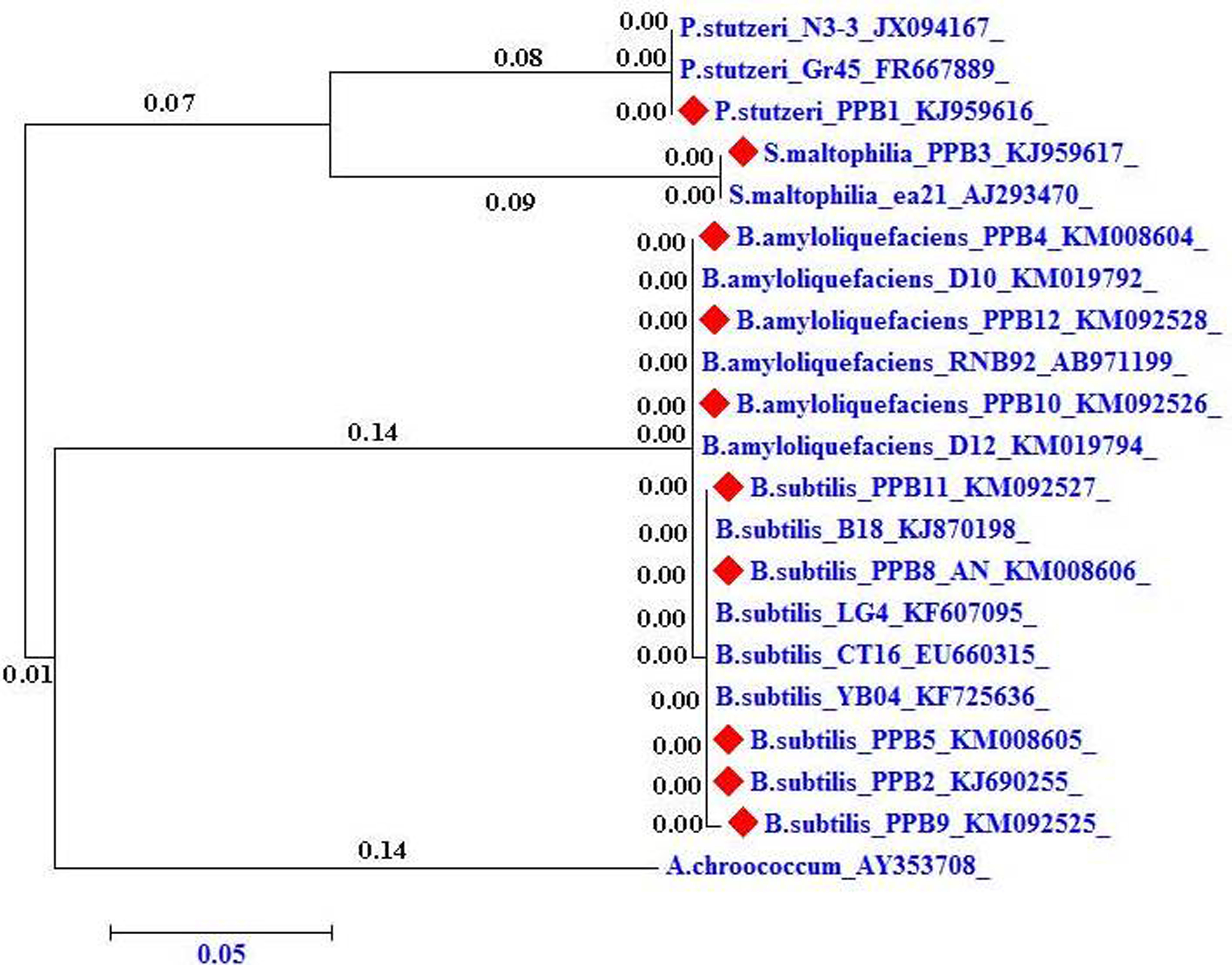

Supplement: FIGURE S1 — | Phylogenetic tree of 16S rRNA gene sequences showing the relationships among the isolates isolated from cucumber rhizosphere. The data of type strains of related species were from GenBank database. [file Image_1.JPEG]
