# Supplementary material for: Isolation and Identification of Plant Growth Promoting Rhizobacteria from Cucumber Rhizosphere and Their Effect on Plant Growth Promotion and Disease Suppression
Source: Front Microbiol. 2016 Feb 2;6:1360. doi: 10.3389/fmicb.2015.01360 (PMC4735380; doi:10.3389/fmicb.2015.01360)
Supplement: Supplementary file 2 [file Table_1.DOCX]

**Table 1 Morphological characteristic of the endophytic bacterial isolates**

| **Strains** | **Colony size and shape** | **Colony color** | **Cell motility** | **Cell shape** |
| --- | --- | --- | --- | --- |
| PPB 1 | Adherent, wrinkled | Reddish brown | Highly motile | Small rods |
| PPB 2 | Small, wavy | Off-white | Highly motile | Medium rods |
| PPB 3 | Glistening, irregular | Greenish white | Motile | Small rods |
| PPB 4 | Small, round | Milky white | Motile | Medium rods |
| PPB 5 | Small, wavy | Reddish | Motile | Medium rods |
| PPB 8 | Medium, round | Milky white | Highly motile | Medium rods |
| PPB 9 | Large, wavy | Off-white | Highly motile | Small rods |
| PPB 10 | Large, round | Milky white | Motile | Medium rods |
| PPB 11 | Medium, wavy | White | Motile | Medium rods |
| PPB 12 | Small, wavy | Milky white | Motile | Medium rods |
